# Colorectal cancer survivors only marginally change their overall lifestyle in the first 2 years following diagnosis

**DOI:** 10.1007/s11764-019-00812-7

**Published:** 2019-10-23

**Authors:** Moniek van Zutphen, Hendriek C. Boshuizen, Dieuwertje E. Kok, Harm van Baar, Anne J. M. R. Geijsen, Evertine Wesselink, Renate M. Winkels, Henk K. van Halteren, Johannes H. W. de Wilt, Ellen Kampman, Fränzel J. B. van Duijnhoven

**Affiliations:** 1 Division of Human Nutrition and Health, Wageningen University & Research, Stippeneng 4, PO Box 17 6708, WE Wageningen, the Netherlands; 2 Department Public Health Sciences, College of Medicine, Penn State University, 500 University Drive, Hershey, PA 17033 USA; 3 Department of Internal Medicine, Admiraal de Ruyter Ziekenhuis, ‘s-Gravenpolderseweg 114, 4462 RA Goes, the Netherlands; 4 Department of Surgery, Radboud University Medical Centre, Geert Grooteplein-Zuid 22, 6525 GA Nijmegen, the Netherlands

**Keywords:** Colorectal cancer, Survivorship, Lifestyle changes, Dietary changes, Lifestyle recommendations

## Abstract

**Purpose:**

A healthy lifestyle after colorectal cancer (CRC) diagnosis may improve prognosis. Data related to lifestyle change in CRC survivors are inconsistent and potential interrelated changes are unknown.

**Methods:**

We assessed dietary intake, physical activity, body mass index (BMI), waist circumference, and smoking among 1072 patients diagnosed with stages I–III CRC at diagnosis, 6 months and 2 years post-diagnosis. An overall lifestyle score was constructed based on the 2018 World Cancer Research Fund/American Institute of Cancer Research recommendations (range 0–7). We used linear mixed models to analyze changes in lifestyle over time.

**Results:**

Participants had a mean (± SD) age of 65 ± 9 years and 43% had stage III disease. In the 2 years following CRC diagnosis, largest changes were noted for sugary drinks (− 45 g/day) and red and processed meat intake (− 62 g/week). BMI (+ 0.4 kg/m^2^), waist circumference (+ 2 cm), and dietary fiber intake (− 1 g/day) changed slightly. CRC survivors did not statistically significant change their mean intake of fruits and vegetables, alcohol, or ultra-processed foods nor did they change their physical activity or smoking behavior. Half of participants made simultaneous changes that resulted in improved concordance with one component as well as deteriorated concordance with another component of the lifestyle score. Overall lifestyle score changed from a mean 3.4 ± 0.9 at diagnosis to 3.5 ± 0.9 2 years post-diagnosis.

**Conclusions:**

CRC survivors hardly improve their overall lifestyle after diagnosis.

**Implications for Cancer Survivors:**

Given the importance of a healthy lifestyle, strategies to effectively support behavior changes in CRC survivors need to be identified.

## Introduction

Rates of cancer survival are increasing, with more people living with and beyond cancer, especially colorectal cancer [1]. Lifestyle recommendations for cancer survivors are largely extrapolated from recommendations for cancer prevention [2]. Cancer survivors who adhere to these recommendations may improve their prognosis. In colorectal cancer (CRC) survivors, for instance, emerging evidence suggests that being physically active or eating a healthy diet after diagnosis may improve survival [3]. However, many CRC survivors show low concordance with these lifestyle recommendations [4–6] and only few receive lifestyle advice [7, 8].

Several, but not all, studies suggest that CRC survivors generally improve specific health behaviors after diagnosis. Retrospective studies suggest these include eating more healthy [9–12], increasing physical activity [11], and quitting smoking [11]. Also, some prospective studies report changes in concordance with lifestyle recommendations after CRC diagnosis, including an increase in vegetable consumption [13–15], an increase in physical activity [13], a decrease in alcohol intake [14], and quitting smoking [15]. In contrast, some prospective studies did not report notable changes in health behaviors after CRC diagnosis—including physical activity [15], alcohol intake [15], or body mass index (BMI) [13]—or even reported changes not in concordance with lifestyle recommendations, such as a decrease in physical activity [16].

Although several studies reported on changes in health behaviors after CRC diagnosis, no studies have examined how these changes are interrelated and few studies tracked behaviors over a 2-year period. Cancer survivors may be inclined to make changes in more than one health behavior [13], but it is unknown whether these changes are correlated with each other. Furthermore, it remains unknown how changes in specific health behaviors impact overall concordance with lifestyle recommendations. The present prospective study aimed to assess changes in health behaviors and overall lifestyle in the first 2 years following CRC diagnosis. We analyzed changes in overall lifestyle by assessing concordance with the World Cancer Research Fund/American Institute for Cancer Research (WCRF/AICR) recommendations. Furthermore, we characterized interrelationships between changes in health behaviors.

## Methods

### Study design and population

We used data from the COLON study, an ongoing prospective multicenter cohort study among CRC patients [17]. From 2010 onwards, newly diagnosed patients with colon or rectal cancer were recruited in 11 hospitals in the Netherlands. Hospital staff invited eligible patients during a routine clinical visit before scheduled surgery. Patients were not eligible when they had a history of CRC, a previous (partial) bowel resection, known hereditary CRC, inflammatory bowel disease, dementia or another mental condition limiting their ability to fill out surveys, or were non-Dutch speaking. Data were collected at baseline (shortly after diagnosis, before treatment started) and at 6 months and 2 years after diagnosis. All study participants provided written informed consent and the study was approved by the local review board.

This study was performed using data of all participants diagnosed with stages I–III CRC between 2010 and 2015 (*n* = 1241). Participants were excluded when information on lifestyle was available for < 2 time points (*n* = 169). Thus, data of 1072 participants remained for analyses. Patients with stage IV disease were excluded a priori, because survival for these patients is generally poor and changes in diet and lifestyle may reflect poor health.

### Data collection

Habitual dietary intake was assessed with a 204-item semi-quantitative food frequency questionnaire (FFQ) at baseline and 6 months and 2 years after CRC diagnosis. The FFQ was developed by the Division of Human Nutrition and Health, Wageningen University & Research, the Netherlands. The reference period for the FFQ was the month before diagnosis at baseline and the previous month during follow-up. To assess amounts of food intake, we combined frequencies of intake with standard portion sizes and household measures [18]. The FFQ was previously validated [19] and slightly adapted to be able to distinguish meat intake with respect to red, processed, and white meat. Self-reported dietary intake data from the FFQ were converted into fiber and alcohol intake based on the 2011 Dutch food composition table [20]. Items of interest included fruits, vegetables, dietary fiber, ultra-processed foods, red and processed meat, sugary drinks, and alcohol.

In addition to the FFQ, participants filled out other lifestyle questionnaires. These questionnaires included questions on weight, waist circumference, physical activity, and smoking status. Patients reported weight at diagnosis and at 6 months and 2 years after diagnosis, while height was only reported at diagnosis. BMI was computed in kg/m^2^. Waist circumference (midway between the lowest rib and the iliac crest) was measured with a tape sent to participants. Moderate-to-vigorous physical activity was self-reported by the validated SQUASH questionnaire [21–23]. Moderate-to-vigorous physical activity included all activities (walking, cycling, gardening, odd jobs, sports, household activities, and work) with a metabolic equivalent value ≥ 3 [24]. To ensure quality of the data, we checked each questionnaire after completion and contacted participants by telephone for clarification if needed.

Information was obtained on demographics, side-effects of treatment, and clinical factors. Demographic information, including level of education and living situation, was self-reported at diagnosis. Furthermore, participants reported if they changed their diet before diagnosis due to bowel complaints and if they experienced side-effects of treatment at 6 months and 2 years after diagnosis. Clinical factors were retrieved from the Dutch ColoRectal Audit [25] and included disease stage, tumor site, receipt of neo-adjuvant treatment, stoma placement after surgery, receipt of adjuvant chemotherapy, and presence of comorbidities. Recurrence data (loco-regional or distant recurrence) were retrieved from the medical records by the Netherlands Cancer Registry.

### WCRF/AICR lifestyle score

We quantified the degree of concordance between participants’ lifestyles and the 2018 WCRF/AICR recommendations for cancer prevention using the standard WCRF/AICR score developed by Shams-White et al. [26] as a measure of overall lifestyle. The score included 7 recommendations (Table 1), as the recommendation on breastfeeding was not applicable to our study population. The recommendations about dietary supplement use and cancer survivors were not included, since they were not operationalized in the standard WCRF/AICR score [26]. We assigned, for each component, 1 point when the recommendation was met (full concordance), 0.5 points when it was partially met (moderate concordance), and 0 points otherwise (low concordance). Quantitative criteria were used as cut-off points, except for the recommendation on ultra-processed foods where cut-offs were based on tertiles calculated as a percentage of total energy intake from ultra-processed foods. Two recommendations (healthy weight and diet rich in wholegrains, vegetables, fruit, and beans) included sub-recommendations. For these recommendations, the recommendation score was the sum of each sub-recommendation score (meaning that plausible scores were 0, 0.25, 0.5, 0.75, and 1). The overall score ranged from 0 to 7, with higher scores indicating greater concordance with the 2018 WCRF/AICR recommendations.

**Table 1:** Description of the standardized WCRF/AICR score based on the 2018 WCRF/AICR recommendations for cancer prevention

WCRF/AICR recommendations	Goal	Operationalization^a^	Scoring
(1) Be a healthy weight.^b^	(1a) Ensure that body weight during childhood and adolescence projects towards the lower end of the healthy adult BMI range	Not operationalized.	-
	(1b) Keep your weight as low as you can within the healthy range throughout life	BMI (in kg/m^2^) 18.5–24.9 BMI 25 to < 30 BMI < 18.5 or ≥ 30	0.5 0.25 0
	(1c) Avoid weight gain (measured as body weight or waist circumference) throughout adulthood	WC men < 94 cm WC women < 80 cm	0.5
		WC men 94 to < 102 cm WC women 80 to < 88 cm	0.25
		WC men ≥ 102 cm WC women ≥ 88 cm	0
(2) Be physically activity.	(2a) Be at least moderately physically active and follow or exceed national guidelines	MVPA ≥ 150 min/week MVPA 75 to < 150 min/week MVPA <75 min/week	1 0.5 0
	(2b) Limit sedentary habits	Not operationalized.	-
(3) Eat a diet rich in wholegrains, vegetables, fruit and beans.^b^	(3a) Consume a diet that provides at least 30 grams per day of fiber from food sources	Dietary fiber intake ≥ 30 g/day Dietary fiber intake 15 to < 30 g/day Dietary fiber intake < 15 g/day	0.5 0.25 0
	(3b) Include in most meals foods containing wholegrains, non-starchy vegetables, fruit, and pulses (legumes) such as beans and lentils.	Not operationalized.	-
	(3c) Eat a diet high in all types of plant foods including ≥ 5 portions/servings (≥ 400 g) of a variety of non-starchy vegetables and of fruit every day	F&V intake ≥ 400 g/day F&V intake 200 to < 400 g/day F&V intake < 200 g/day	0.5 0.25 0
	(3d) If you eat starchy roots and tubers as staples, eat non-starchy vegetables, fruit, and pulses (legumes) regularly too if possible	Not operationalized.	-
(4) Limit consumption of “fast foods” and other processed foods high in fat, starches, or sugars.	(4a) Limit consumption of processed foods high in fat, starches or sugars—including “fast foods”; many pre-prepared dishes, snacks, bakery foods, and desserts; and confectionary (candy)	Ultra-processed foods T1 (≤ 23.7 en%) Ultra-processed foods T2 (23.7 to ≤ 32.0 en%) Ultra-processed foods T3 (> 32.0 en%)	1 0.5 0
(5) Limit consumption of red and processed meat.	(5a) If you eat red meat, limit consumption to no more than about three portions per week. Three portions is equivalent to about 350 to 500 grams cooked weight of red meat. Consume very little, if any, processed meat	Red meat ≤ 500 g/week and processed meat intake < 21 g/week	1
		Red meat ≤ 500 g/week and processed meat intake 21 to < 100 g/week	0.5
		Red meat and processed meat > 500 g/week or processed meat intake ≥ 100 g/week	0
(6) Limit consumption of sugar sweetened drinks.	(6a) Do not consume sugar-sweetened drinks	Sugary drink intake 0 g/day Sugary drink intake ≤ 250 g/day Sugary drink intake > 250 g/day	1 0.5 0
(7) Limit alcohol consumption.	(7a) For cancer prevention, it’s best not to drink alcohol	Alcohol intake 0 g/day	1
		Alcohol intake men ≤ 20 g/day (2 drinks) Alcohol intake women ≤ 10 g/day (1 drink)	0.5
		Alcohol intake men > 20 g/day (2 drinks) Alcohol intake women > 10 g/day (1 drink)	0
(8) Do not use supplements for cancer prevention.	(8a) High-dose dietary supplements are not recommended for cancer prevention—aim to meet nutritional needs through diet alone	Not operationalized.	-
(9) For mothers: breastfeed your baby, if you can.	(9) This recommendation aligns with the advice of the World Health Organization, which recommends infants are exclusively breastfed for 6 months, and then up to 2 years of age or beyond alongside appropriate complementary foods	Not applicable to this population	-
(10) After a cancer diagnosis: follow our recommendations, if you can.	(10a) All cancer survivors should receive nutritional care and guidance on physical activity from trained professionals.	Not operationalized.	-
	(10b) Unless otherwise advised, and if you can, all cancer survivors are advised to follow the Cancer Prevention Recommendations as far as possible after the acute stage of treatment.	Not operationalized.	-

*BMI*, body mass index; *en%*, energy percentage; *F&V*, fruit and vegetables; *MVPA*, moderate-to-vigorous physical activity; *T*, tertile; *WC*, waist circumference; *WCRF/AICR*, World Cancer Research Fund/American Institute for Cancer Research

^a^Ultra-processed foods included French fries, crisps, pastry and biscuits, savory snacks, sugar and candy, sauces, pizza, pancake, sandwich fillings high in sugar or fat, refined grain products, and sweet dairy desserts. Not included were yoghurt and cheese, nuts, oils and fats, sugary drinks, processed meat, and diet soft drinks. Calculated as energy intake from ultra-processed foods of total energy intake. Sugary drinks included sugar-sweetened soft drinks, sugar-sweetened dairy drinks, and fruit juices.

^b^The score for recommendations 1 and 3 was the result of summing the scores of each sub-recommendation

### Statistical analyses

To describe the study population, we used descriptive analyses of demographic, clinical, and lifestyle characteristics of the participants. Furthermore, we calculated concordance with the 7 WCRF/AICR recommendations at diagnosis and 6 months and 2 years after diagnosis.

To describe changes over time in health behaviors in the first 2 years after CRC diagnosis, we used linear mixed models. Linear mixed models take into account both the individual trajectories of change and population averages by using all available measurements and including participants with incomplete data [27]. Each health behavior was modelled separately by using the 3 repeated measurements of that dependent variable. Time was scaled in years (continuous) and calculated as date of survey completion minus the date of study enrolment (i.e., shortly after diagnosis). All models included a random intercept, while a random slope was only included when this resulted in a better fit of the model (i.e., for BMI and ultra-processed foods). Inclusion of a random slope in the model means that the change over time can vary between participants. Changes were considered to be in concordance with lifestyle recommendations when the changes were as follows: an increase in physical activity, dietary fiber, fruit and vegetable intake or a decrease in BMI, waist circumference, red and processed meat, ultra-processed foods, sugary drinks, or alcohol intake.

To assess if multiple changes in different health behaviors led to a change in overall lifestyle, we modelled the 3 repeated measures of the WCRF/AICR lifestyle score as a dependent variable in a linear mixed model with random slope (in the same way as described above). To assess if changes in overall lifestyle varied between subgroups, we included a grouping factor and its interaction term with time in the mixed models. As grouping factors, baseline demographic determinants (sex, age, education, and living situation), clinical characteristics (stage, tumor site, stoma, neo-adjuvant treatment, adjuvant chemotherapy, and comorbidities) and self-reported side-effects of treatment were included, each in a separate model.

To further assess the interrelatedness between changes in multiple health behaviors, we assessed change in concordance to the 7 components of the WCRF/AICR lifestyle score. We assessed the proportion of participants who did change concordance to ≥ 1 component(s), who only improved or only deteriorated concordance to ≥ 1 component(s), and who both improved and decreased concordance to components of the lifestyle score. Furthermore, we assessed Pearson correlations between changes in health behaviors.

By using two separate sensitivity analyses, we evaluated the robustness of our reported changes in lifestyle. The potential influence of recurrent CRC or pre-diagnosis illness on changes in lifestyle was determined by excluding participants diagnosed with a recurrence within 2 years of follow-up (*n* = 98) and by excluding those who reported pre-diagnosis changes in diet due to bowel complaints (*n* = 129), respectively. All statistical analyses were conducted using SAS 9.4 software (SAS Institute, Cary NC). A *p* value < 0.05 was considered statistically significant.

## Results

### Study population

Participants had a mean ± SD age of 65 ± 9 years, 63% was male, 67% had colon cancer, and 11% was a current smoker at diagnosis (Table 2). Stage III disease was more common (43%) than stage II (30%) or stage I disease (26%).

**Table 2:** Baseline demographic, clinical, and lifestyle characteristics

	Total
*N*	1072
Age at diagnosis (mean ± SD) (years)	65 ± 9
Men (%)	680 (63%)
Education levela	
Low	463 (43%)
Medium	263 (25%)
High	342 (32%)
Living with partner^a^	903 (84%)
Tumor stage	
I	284 (26%)
II	325 (30%)
III	463 (43%)
Tumor site	
Colon	719 (67%)
Rectum	353 (33%)
Neo-adjuvant therapy (%)	258 (24%)
Adjuvant chemotherapy (%)^b^	258 (24%)
Stoma (%)^b^	312 (29%)
Experienced side-effects of treatment (6 months after diagnosis)^a^	689 (65%)
Experienced side-effects of treatment (2 years after diagnosis)^c^	500 (53%)
Comorbidity at diagnosis (%)^a^	709 (66%)
Current smoker at diagnosis (%)^a^	116 (11%)
BMI (kg/m^2^)	
< 18.5	8 (1%)
18.5–25	411 (38%)
25–30	469 (44%)
30–35	150 (14%)
> 35	34 (3%)

Education level: low, primary, and pre-vocational; medium, secondary, and vocational; high, university.

^a^Data of 3 to 10 participants were missing/unknown

^**b**^Data of 23 to 29 participants were missing/unknown

^c^Data of 124 participants were missing/unknown

### Concordance with lifestyle recommendations

**Fig. 1:**
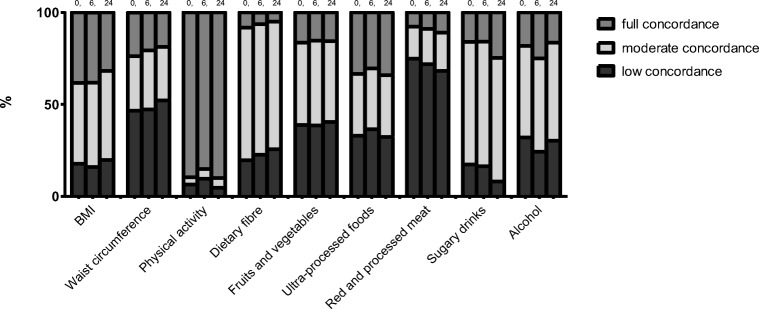
Concordance with the 2018 World Cancer Research Fund/American Institute for Cancer Research lifestyle recommendations at 0, 6, and 24 months after colorectal cancer diagnosis

Participants showed large variation in their concordance with the WCRF/AICR lifestyle recommendations (Fig 1). Upon CRC diagnosis, few participants reported full concordance with the dietary recommendations. The lowest concordance was observed for the recommendation to limit intake of red and processed meat (8%) and the highest concordance was observed for the recommendation to limit intake of ultra-processed foods (33%). In contrast, the majority of patients (90%) adhered to the physical activity recommendation at CRC diagnosis. Furthermore, 38% of patients had a BMI within the healthy range and 24% had a healthy waist circumference.

### Change in health behaviors

Some changes in concordance with the WCRF/AICR lifestyle recommendations were seen in the first 2 years after diagnosis for specific health behaviors (Table 3). Most improvement were noted for sugary drinks (− 45 g/day) and red and processed meat intake (− 62 g/week). Changes not in concordance with the recommendations were the decrease in fiber intake (1 g/day) and the increase in BMI (0.4 kg/m^2^) and waist circumference (2 cm). On average, participants did not change their intake of fruit and vegetables, ultra-processed foods nor did they change their smoking behavior (*p* > 0.05). Participants initially decreased their intake of alcohol and their physical activity level in the first 6 months after diagnosis. Although alcohol intake and physical activity levels were still lower 2 years after diagnosis compared with diagnosis, these decreases were not statistically significant.

**Table 3:** Changes in lifestyle in the first 2 years after colorectal cancer diagnosis, sorted by effect size (*n* = 1072).

	Baseline^a^	6 months after diagnosis	2 years after diagnosis	Change from diagnosis to 2 years after diagnosis^b^	Effect size^c^	*p* trend^d^
*n*	1066	1056	931			
WCRF/AICR score	3.4 ± 0.9	3.4 ± 0.9	3.5 ± 0.9	0.1	0.10	< 0.001
Sugary drinks (g/day)^e^	128 ± 165	127 ± 161	83 ± 120	-45	− 0.30	< 0.001
Red & processed meat (g/week)	485 ± 263	455 ± 257	423 ± 255	-62	− 0.24	< 0.001
Dietary fiber (g/day)	20 ± 7	20 ± 6	19 ± 6	-1	− 0.15	< 0.001
Physical activity (min/week)	852 ± 723	621 ± 566	766 ± 650	-86	− 0.13	0.16
Waist circumference (cm)	96 ± 12	96 ± 12	98 ± 12	2	0.11	< 0.001
BMI (kg/m^2^)	26.5 ± 4.0	26.4 ± 3.8	26.9 ± 4.0	0.4	0.09	< 0.001
Alcohol (g/day)	14 ± 17	11 ± 14	13 ± 14	-1	− 0.08	0.06
Fruit & vegetables (g/day)	261 ± 147	258 ± 142	258 ± 145	-3	− 0.03	0.38
Ultra-processed foods (en%)^f^	28.4 ± 10.4	28.9 ± 10.1	28.2 ± 10.5	-0.2	− 0.02	0.10
Current smoker (%)	11%	8%	9%	-2	− 0.09	0.19

^a^Mean ± SD (all such values)

^b^Year 2-baseline

^c^Effect size = 2-year change/pooled SD

^d^*p* trend values were based on linear mixed models that included the three repeated measurements. For smoking, a logistic mixed model was used

^e^Sugary drinks included sugar-sweetened soft drinks, sugar-sweetened dairy drinks, and fruit juices

^f^Ultra-processed foods included French fries, crisps, pastry and biscuits, savory snacks, sugar and candy, sauces, pizza, pancake, sandwich fillings high in sugar or fat, refined grain products, and sweet dairy desserts. Not included were yoghurt and cheese, nuts, oils and fats, sugary drinks, processed meat, and diet soft drinks. Intake of ultra-processed foods was shown in energy percentage (energy intake ultra-processed foods/total energy intake × 100%).

### Interrelationships between changes

Although participants changed some health behaviors, overall lifestyle improved only marginally. Overall lifestyle changed from a mean (± SD) 3.4 ± 0.9 at diagnosis to 3.5 ± 0.9 2 years later (*p* < 0.001) (Table 3). Two-year changes in overall lifestyle did not statistically significant differ between subgroups based on demographics (sex, age, education), clinical characteristics (stage, tumor site, treatment, comorbidities), or self-reported side effects of treatment (data not shown). The only difference between subgroups was noted for living situation. Participants living without a partner had a better 2-year improvement in overall lifestyle (+ 0.2) than participants living with their partner (+ 0.1, *p*_interaction_ = 0.04), while overall lifestyle was similar at diagnosis.

Almost all participants (92%) changed concordance with at least 1 of the 7 WCRF/AICR lifestyle recommendations in the first 2 years after CRC diagnosis. Seventy percent of participants improved concordance with at least 1 recommendation. About half (51%) of participants made simultaneous changes that resulted in both improved concordance with ≥ 1 component and deteriorated concordance with another component of the lifestyle score. Furthermore, 20% of participants only improved their concordance and 24% only decreased their concordance.

**Fig. 2:**
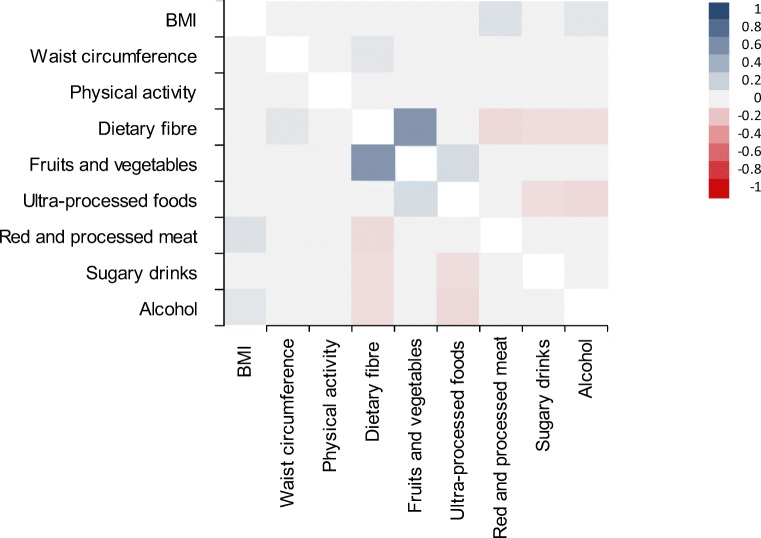
Pairwise correlations for changes in health behaviors included in the 2018 World Cancer Research Fund/American Institute for Cancer Research score in the first 2 years following a colorectal cancer diagnosis. A blue square represents a positive correlation in which both changes go in the same direction. A red square represents an inverse correlation in which one change is in line with the recommendations and the other is not. The darker the color, the stronger the correlation. A grey square represents a non-significant correlation (*p* > 0.05).

Although many participants made simultaneous changes, participants did not show a clear pattern of changes in health behaviors (Fig. 2). Correlations between 2-year changes in health behaviors ranged from *r* = − 0.11 to *r* = 0.14. An exception was seen for the correlation between changes in dietary fiber and fruits and vegetable intake (*r* = 0.56).

### Sensitivity analyses

No differences in effect sizes were observed after excluding participants who reported to have made pre-diagnosis changes in diet due to bowel complaints (*n* = 129), although the decrease in physical activity and alcohol intake became statistically significant (*p* = 0.05 and *p* = 0.04, respectively; data not shown). The effect sizes also did not differ when we excluded participants diagnosed with a recurrence within 2 years after diagnosis (*n* = 98), although the decrease in ultra-processed foods became statistically significant (*p* = 0.05).

## Discussion

In this prospective observational study, CRC survivors with stages I–III disease only marginally changed their overall lifestyle in the first 2 years after CRC diagnosis. Lifestyle was not in concordance with many of the WCRF/AICR lifestyle recommendations for cancer prevention during that period. Largest changes were noted for sugary drinks and red and processed meat intake. These improvements did not necessarily lead to a higher overall lifestyle score, as half of participants made simultaneous changes that resulted in both improved concordance with one component and deteriorated concordance with another component of the lifestyle score.

The current study was the first that characterized interrelationships between health behavior changes after CRC diagnosis. Overall lifestyle, as reflected by the 2018 WCRF/AICR score, only changed marginally from 3.4 at diagnosis to 3.5 2 years after diagnosis. No differences in lifestyle changes were observed by clinical characteristics—such as stage, tumor site, treatment, or presences of comorbidities—, demographics, or self-reported side-effects of treatment. The only difference between subgroups was that participants living without a partner made slightly larger improvements to their overall lifestyle compared with those living with their partner. The overall improvement of 0.1 on the 7-point scale is probably not relevant, as it is an improvement of only 1%. Although almost all participants (92%) changed concordance with at least one WCRF/AICR lifestyle recommendation, participants did not show a clear pattern of simultaneous changes in health behaviors.

As this was the first study that examined changes in overall lifestyle in CRC patients, we can only compare our results on changes in specific health behaviors with previous studies in CRC patients. The largest observed change in our study was a decrease in sugary drink intake by 45 g/day, equivalent to a decrease of 2 servings (2 × 150 g) per week. Ours was the first study that assessed changes in sugary drink intake after CRC diagnosis. The second largest observed change was a decrease in red and processed meat intake by 62 g/week. This is equivalent to, for example, a combined decrease of 0.3 serving (0.3 × 100 g) of red meat per week and 2 servings of processed meat (2 × 16 g as sandwich filling) per week and is in line with previous prospective studies [14, 15].

Changes not in line with the lifestyle recommendations were the slight decrease in fiber intake (1 g/day) and the slight increase in BMI (0.4 kg/m^2^) and waist circumference (2 cm). Also, several other studies have reported that weight gain after diagnosis is common among CRC patients [15, 28–32]. However, we previously concluded that post-diagnosis weight gain was mainly observed in individuals who lost weight before CRC diagnosis and post-diagnosis weight was similar to pre-diagnosis weight in this study population [33]. Participants did not change their intake of ultra-processed foods or fruit and vegetables, while the intake of alcohol and levels of physical activity tended to decline, especially in the first 6 months after diagnosis. Although previous prospective studies have shown an increase in vegetable intake after CRC diagnosis [13–15], results for changes in other health behaviors are inconsistent between studies [13–16, 34]. Together, these results suggest that CRC survivors improve some health behaviors after diagnosis, but other health behaviors may worsen after CRC diagnosis.

Overall, our findings provide little evidence that a CRC diagnosis triggers desirable lifestyle changes over and above lifestyle trends in the general adult population. Participants showed encouraging trends over time in sugary drinks and red and processed meat intake, in line with general health and nutrition advice. However, these trends have also been noted in the general Dutch adult population [35]; the intake of sugary drinks decreased with 49 g/day and the intake of red and processed meat decreased with 42 g/week in the period between 2012 and 2016. Furthermore, two previous studies have concluded that changes in health behaviors did not differ between CRC survivors and people without a cancer diagnosis [13, 15]. Together, these results suggest that changes in lifestyle after a cancer diagnosis may not be particularly related to the cancer diagnosis.

Both the lack of improvement in overall lifestyle and the discrepancy between lifestyle guidelines and the practiced lifestyle behaviors indicate that lifestyle support is needed after CRC diagnosis. Previous studies [4–6, 36] also reported only moderate concordance with lifestyle recommendations at cancer diagnosis and thereafter, leaving room for improvement in different lifestyle behaviors. Although there is growing evidence that healthier lifestyles after diagnosis are important for CRC outcomes, the evidence that changing these behaviors would alter the clinical course of CRC is limited [2, 3]. However, the current understanding of cancer and its relations with diet and physical activity supports the idea that cancer survivors should change their behavior in concordance to the WCRF/AICR lifestyle recommendations to improve their long-term outcomes [2]. Therefore, support and guidance for a healthy diet and physical activity should be included as part of cancer survivorship care [2, 37]. Few of our participants received guidance on a healthy lifestyle, as is currently the case for most cancer survivors [7, 8]. Research is needed to evaluate the most effective support and to define the benefits of lifestyle changes in cancer survivors.

Given the probable improvement in prognosis with a healthy lifestyle, it is important that healthcare providers discuss lifestyle behaviors with their cancer patients. Three actions appear to be key steps in interventions to support a healthy lifestyle: asking, advising, and arranging, especially for the oncologist [38]. For example, the oncologist could ask how many minutes per week do you do exercise. If the answer is 150 or more, the oncologist can provide positive reinforcement; if not, the oncologist can advise to strive to do so and arrange referral to a trained exercise professional when needed. Using this approach, the oncologist can initiate and reinforce behavior change, but a trained professional should oversee and support the process of behavior change.

Potential limitations of our study should be considered. Diet and lifestyle were self-reported at each time point; thus, only people who were motivated to fill out such questionnaires were included. This could potentially limit generalizability of the results. However, ranges of dietary intakes, physical activity, and BMI were broad and overlapped with national estimates [39–41] and CRC survivors not interested to participate in the study are unlikely to make more or larger improvements in lifestyle. Furthermore, self-reporting might lead to measurement error with regard to lifestyle changes. Generally, systematic errors are present in self-reported lifestyle data; some people underreport, while others overreport. However, participants are likely to have internal consistency in their reporting [42]. Therefore, changes in lifestyle may be less prone to such bias than single lifestyle measurements. Second, a large part of our study population (90%) was active at or over the recommended 150 min/week. This is slightly higher than the general Dutch population aged 65–80 years, in which 76% meets the physical activity recommendation [43]. However, this activity level was similar to the 91% concordance to the physical activity guideline that was found in another study among Dutch CRC survivors [10]. The lack of increase in physical activity might be due to our active study population, since an increase in physical activity has been observed before in CRC survivors in the USA [13], where the proportion meeting the activity recommendation is much lower. Similarly, our study population contained few current smokers at diagnosis (11%), which might explain a lack of decrease in smoking. Third, we assumed that diet and lifestyle at diagnosis represents usual pre-diagnosis diet and lifestyle although these might have been altered because of illness. However, no differences in changes in overall lifestyle and specific health behaviors were observed after excluding participants who reported to have made pre-diagnosis changes in diet due to bowel complaints. Fourth, disease recurrence may influence lifestyle. However, when we excluded participants diagnosed with a recurrence within 2 years after diagnosis, our results did not change. Another limitation might be the potential influence of side-effects of treatment on lifestyle. Those side-effects are more likely to impact lifestyle at 6 months after diagnosis than 2 years after diagnosis, as chemotherapy is usually not completed within 6 months after diagnosis and also recovery from surgery might not be complete yet. Therefore, we focused our analyses on 2-year changes, while still taking 6-month changes into account. Two-year changes represent relatively long-term changes that are sustained over prolonged time. These long-term changes are more likely to impact cancer outcomes than short-term changes and are therefore considered the most relevant changes.

This study has several strengths. First, the COLON study provided an opportunity to prospectively study changes in multiple health behaviors and overall lifestyle in the first 2 years after diagnosis. We used mixed models to examine these changes after CRC diagnosis. An advantage of mixed models is that participants with incomplete lifestyle data during follow-up were still included in the analyses. Second, we had detailed clinical information available and we were thus able to compare lifestyle changes between different subgroups. No differences in lifestyle changes were observed by clinical characteristics, such as stage or tumor site.

In conclusion, our results show that overall lifestyle only marginally changed in the 2 years following CRC diagnosis. Future studies are needed to confirm our findings and to assess how post-diagnosis changes in lifestyle relate to recurrence, survival, and the development of comorbidities. The growing evidence that healthier lifestyles are important for long-term cancer outcomes [3] highlights the need for strategies to effectively support health behavior change in CRC survivors.

## Data Availability

The data that support the findings of this study are available from the corresponding author upon reasonable request.
